# Supplementary material for: Predictive Value of Baseline [18F]FDG PET/CT for Response to Systemic Therapy in Patients with Advanced Melanoma
Source: J Clin Med. 2021 Oct 27;10(21):4994. doi: 10.3390/jcm10214994 (PMC8584809; doi:10.3390/jcm10214994)
Supplement: Supplementary file 1 [file jcm-10-04994-s001.zip › jcm-1428994-supplementary.pdf]

SUPPLEMENTAL MATERIAL – FIGURES LEGEND

Figure S1. Study workflow.

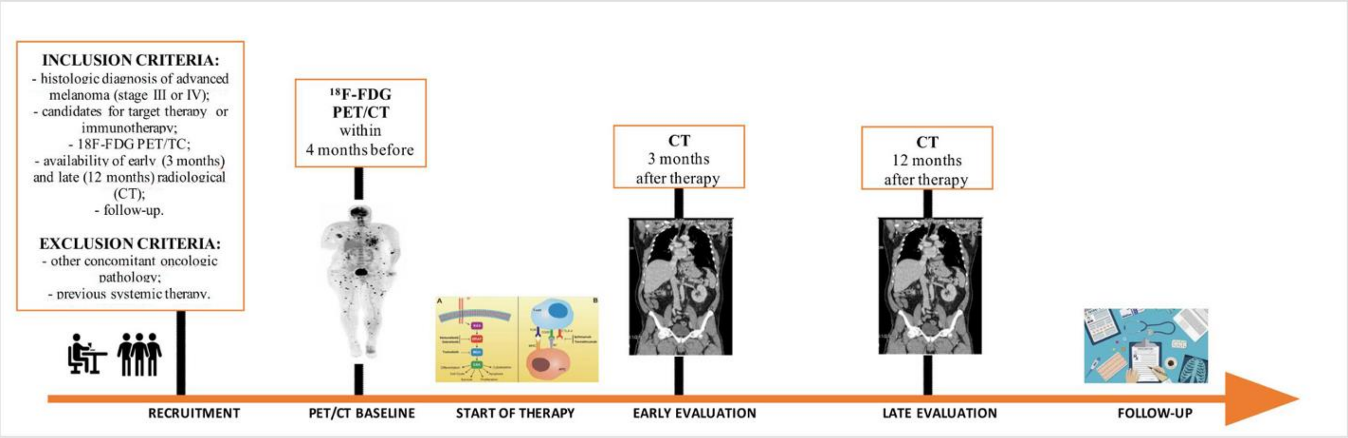

Figure S2. Optimal cut-offs of semi-quantitative parameters to predict responder vs non-responder patients at 12 months defined using the receiver operating characteristic (ROC) curve.

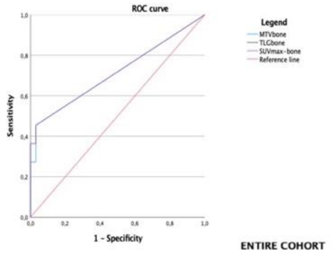

Parameters at 12 months	Optimal cut-off	Sensitivity	Specificity	AUC	p-value
**Entire cohort**					
Bone MTV (mL)	6.1	36%	97%	0.713	0.037
Bone TLG	18.8	45%	97%	0.716	0.034
Bone SUVmax	4.1	36%	97%	0.716	0.034

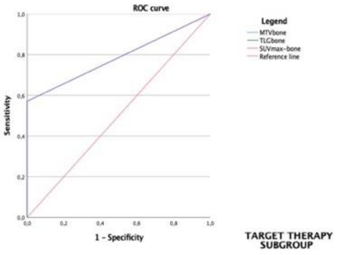

Parameters at 12 months	Optimal cut-off	Sensitivity	Specificity	AUC	p-value
**Target therapy cohort**					
Bone MTV (mL)	13.1	42%	100%	0.786	0.027
Bone TLG	18.8	57%	100%	0.786	0.027
Bone SUVmax	5.7	42%	100%	0.786	0.027

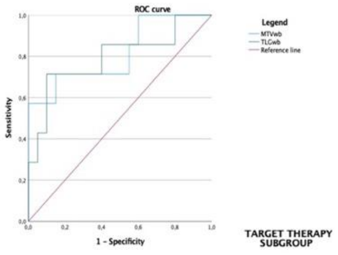

Parameters at 12 months	Optimal cut-off	Sensitivity	Specificity	AUC	p-value
**Target therapy cohort**					
Whole body MTV (mL)	24.6	71%	85%	0.814	0.015
Whole body TLG	208.4	71%	85%	0.793	0.023

Figure S3. Optimal cut-offs of semi-quantitative parameters to predict PFS and OS defined using the receiver operating characteristic (ROC) curve.

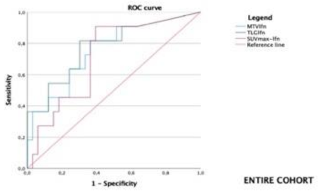

Parameters for OS	Optimal cut-off	Sensitivity	Specificity	AUC	p-value
**Entire cohort**					
Lymph nodes MTV (mL)	10.6	63%	76%	0.755	0.012
Lymph nodes TLG	55.1	63%	76%	0.777	0.006
*Lymph nodes SUVmax**	9.7	72%	64%	0.713	0.036

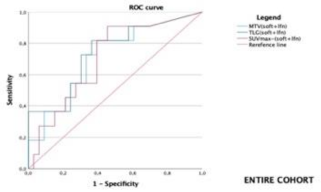

Parameters for OS	Optimal cut-off	Sensitivity	Specificity	AUC	p-value
**Entire cohort**					
Soft tissue +LFN MTV (mL)	10.6	72%	67%	0.713	0.036
Soft tissue + LFN TLG	66.1	63%	70%	0.738	0.019
*Soft tissue + LFN SUVmax**	9.2	81%	61%	0.702	0.046

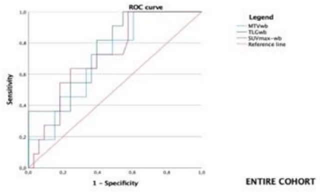

Parameters for OS	Optimal cut-off	Sensitivity	Specificity	AUC	p-value
**Entire cohort**					
Whole body MTV (mL)	14.8	72%	61%	0.705	0.043
Whole body TLG	86.4	72%	64%	0.760	0.010
*Whole body SUVmax**	11.6	72%	61%	0.720	0.030

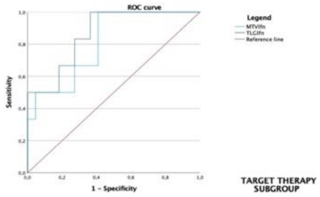

Parameters for OS	Optimal cut-off	Sensitivity	Specificity	AUC	p-value
**Target therapy cohort**					
Lymph nodes MTV (mL)	10.9	66%	73%	0.811	0.022
Lymph nodes TLG	137.4	66%	82%	0.864	0.007

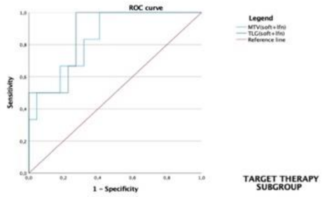

Parameters for OS	Optimal cut-off	Sensitivity	Specificity	AUC	p-value
**Target therapy cohort**					
Soft tissue +LFN MTV (mL)	14.6	66%	82%	0.841	0.012
Soft tissue + LFN TLG	132.1	66%	78%	0.871	0.006

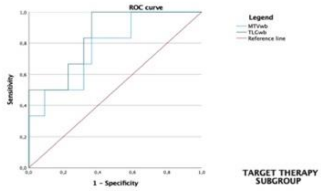

Parameters for OS	Optimal cut-off	Sensitivity	Specificity	AUC	p-value
**Target therapy cohort**					
Whole body MTV (mL)	17.6	66%	69%	0.773	0.044
Whole body TLG	158.1	66%	78%	0.848	0.010

SUPPLEMENTAL MATERIAL – TABLE LEGEND

Table S1. Semi-quantitative parameters extrapolated for each district from the PET images.

**Parameters** mean ± SD (range)	**Entire cohort (44/44)**	**Target therapy cohort (28/44)**	**Immunotherapy cohort (16/44)**
Soft tissue MTV (mL)	5.3 ± 15.3 (0.0 - 77.5)	3.0 ± 9.5 (0.0 - 49.4) **	9.3 ± 21.9 (0.0 – 77.5) **
Soft tissue TLG	25.8 ± 75.4 (0.0 – 336.4)	19.1 ± 66.3 (0.0 – 336.4)	37.7 ± 90.3 (0.0 – 269.5)
Soft tissue SUVmax*	2.9 ± 6.2 (0.0 - 25.6)	3.1 ± 6.0 (0.0 – 25.6)	2.6 ± 6.6 (0.0 – 24.5)
Lymph nodes MTV (mL)	19.2 ± 49.9 (0.0 – 304.4)	25.2 ± 61.2 (0.0 – 304.4)	8.8 ± 14.3 (0.0 – 49.7)
Lymph nodes TLG	133.9 ± 289.3 (0.0 – 1634.5)	166.8 ± 337.6 (0.0 – 1634.5)	76.3 ± 171.1 (0.0 – 682.9)
Lymph nodes SUVmax*	12.1 ± 17.2 (0.0 – 82.3)	14.2 ± 20.1 (0.0 – 82.3)	8.4 ± 9.9 (0.0 – 28.7)
Soft tissue +LFN MTV (mL)	24.6 ± 53.8 (0.0 – 304.4)	28.2 ± 64.8 (0.0 – 304.4)	18.2 ± 25.6 (0.0 – 79.0)
Soft tissue + LFN TLG	159.8 ± 308.3 (0.0 – 1634.5)	185.9 ± 361.5 (0.0 – 1634.5)	114.1 ± 183.8 (0.0 – 682.9)
Soft tissue + LFN SUVmax*	12.6 ± 17.1 (0.0 – 82.3)	14.8 ± 19.9 (0.0 – 82.3)	8.8 ± 10.1 (0.0 – 28.7)
Lung MTV (mL)	2.0 ± 3.7 (0.0 – 16.1)	0.7 ± 2.5 (0.0 – 12.4) **	4.2 ± 4.3 (0.0 – 16.1) **
Lung TLG	10.2 ± 25.5 (0.0 – 143.4)	1.9 ± 7.2 (0.0 – 36.2) **	24.6 ± 37.8 (0.0 – 143.4) **
Lung SUVmax*	2.9 ± 4.9 (0.0 – 18.4)	0.7 ± 2.0 (0.0 – 9.5) **	6.6 ± 6.2 (0.0 – 18.4) **
Liver MTV (mL)	0.7 ± 2.9 (0.0 – 16.76)	0.9 ± 3.6 (0.0 – 16.7)	0.2 ± 1.1 (0.0 – 4.6)
Liver TLG	5.0 ± 25.5 (0.0 – 166.0)	7.2 ± 31.8 (0.0 – 166.0)	1.2 ± 5.1 (0.0 – 20.7)
Liver SUVmax*	1.5 ± 8.9 (0.0 – 59.2)	2.2 ± 11.1 (0.0 – 59.2)	0.3 ± 1.5 (0.0 – 6.3)
Bone MTV (mL)	3.3 ± 10.4 (0.0 – 49.1)	4.0 ± 12.1 (0.0 – 49.1)	2.2 ± 6.5 (0.0 – 24.4)
Bone TLG	12.7 ± 40.9 (0.0 – 236.5)	15.9 ± 49.1 (0.0 – 236.5)	7.2 ± 19.9 (0.0 – 66.0)
Bone SUVmax*	2.2 ± 9.5 (0.0 -61.9)	3.0 ± 11.8 (0.0 – 61.9)	0.8 ± 2.6 (0.0 – 9.8)
Other MTV (mL)	1.9 ± 6.9 (0.0 – 42.7)	1.4 ± 3.6 (0.0 – 15.1)	2.7 ± 10.6 (0.0 – 42.7)
Other TLG	12.8 ± 45.3 (0.0 – 253.6)	10.8 ± 32.2 (0.0 – 131.0)	16.1 ± 63.3 (0.0 – 253.6)
Other SUVmax*	2.2 ± 7.5 (0.0 – 40.3)	2.9 ± 9.1 (0.0 – 40.3)	1.0 ± 3.0 (0.0 – 253.6)
Whole body MTV (mL)	32.7 ± 59.9 (0.8 – 329.5)	35.5 ± 72.0 (0.8 – 329.5)	27.7 ± 30.6 (1.5 – 103.5)
Whole body TLG	198.4 ± 326.2 (1.5 – 1732.9)	218.4 ± 384.4 (1.5 – 1732.9)	163.4 ± 192.5 (5.2 – 682.9)
Whole body SUVmax*	15.5 ± 16.0 (1.2 – 82.3)	16.4 ± 19.4 (1.7 – 82.3)	14.0 ± 7.3 (1.2 – 28.7)

NOTE: LFN = lymph nodes; MTV = metabolic tumor volume, SUVmax= maximum standardize uptake value, TLG = total lesion glycolysis.

* Corresponds to the highest SUVmax found in each district.

** The only statistically different means to Levine's test (*p<0.05*), comparing the means of the two sub-cohorts (target therapy vs immunotherapy).

Table S2. Semi-quantitative parameters extrapolated for each district from the PET images associated to early and late response assessment results.

**Parameters**	**Entire cohort (44/44)**			**Target therapy cohort (28/44)**			**Immunotherapy cohort (16/44)**		
**At 3 months**	Responders (mean)	Non responders (mean)	*p* value	Responders (mean)	Non responders (mean)	*p* value	Responders (mean)	Non responders (mean)	*p* value
Soft tissue MTV (mL)	5.2	5.8	0.881	3.2	0.4	0.936	9.6	8.6	0.935
Soft tissue TLG	22.8	45.2	0.726	20.1	6.6	0.640	28.8	64.6	0.862
Soft tissue SUVmax*	2.4	6.2	0.628	2.4	12.8	0.476	2.5	2.9	0.862
Lymph nodes MTV (mL)	21.9	2.9	0.245	26.9	3.3	0.381	11.0	2.6	1.000
Lymph nodes TLG	149.4	36.0	0.431	173.1	86.1	0.698	98.2	11.0	0.862
Lymph nodes SUVmax*	11.6	15.6	0.777	12.7	34.7	0.894	9.3	6.0	0.862
Soft tissue +LFN MTV (mL)	27.1	.87	0.259	30.1	3.7	0.296	20.6	11.2	1.000
Soft tissue + LFN TLG	172.3	81.3	0.514	193.2	92.6	0.582	127.0	75.7	0.953
Soft tissue + LFN SUVmax*	12.0	16.5	0.881	13.3	34.7	0.963	9.4	7.4	1.000
Lung MTV (mL)	1.9	2.8	0.493	0.9	0.0	0.698	4.2	4.2	0.684
Lung TLG	9.6	14.5	0.451	2.1	0.0	0.698	25.6	21.8	0.684
Lung SUVmax*	2.5	5.4	0.431	0.8	0.0	0.698	6.2	8.1	0.684
Liver MTV (mL)	0.7	0.8	0.701	1.0	0.0	0.894	0.0	1.2	0.521
Liver TLG	5.3	3.5	0.701	7.8	0.0	0.894	0.0	5.2	0.521
Liver SUVmax*	1.7	1.1	0.677	2.4	0.0	0.894	0.0	1.6	0.521
Bone MTV (mL)	2.3	10.1	0.374	2.5	24.6	0.339	2.0	2.8	0.684
Bone TLG	6.8	50.4	0.356	8.1	118.3	0.339	4.1	16.5	0.599
Bone SUVmax*	2.0	3.7	0.356	2.8	6.2	0.381	0.4	2.5	0.599
Other MTV (mL)	2.0	1.2	1.000	1.3	3.6	0.529	3.6	0.0	0.684
Other TLG	14.0	5.0	0.987	10.6	15.0	0.476	21.5	0.0	0.684
Other SUVmax*	2.4	1.2	0.987	2.9	3.5	0.476	1.4	0.0	0.684
Whole body MTV (mL)	34.2	23.6	0.987	35.9	32.0	0.640	30.5	19.4	0.599
Whole body TLG	205.4	154.7	0.582	217.9	225.9	0.296	178.3	119.1	0.684
Whole body SUVmax*	14.6	22.1	0.289	14.6	40.9	0.222	14.5	12.7	0.862
**Parameters**	**Entire cohort (44/44)**			**Target therapy cohort (28/44)**			**Immunotherapy cohort (16/44)**		
**At 12 months**	Responders (mean)	Non responders (mean)	*p* value	Responders (mean)	Non responders (mean)	*p* value	Responders (mean)	Non responders (mean)	*p* value
Soft tissue MTV (mL)	4.6	7.8	0.945	1.6	7.4	0.935	9.6	8.6	0.953
Soft tissue TLG	16.8	54.7	0.924	9.6	49.0	0.893	28.8	64.6	0.862
Soft tissue SUVmax*	3.1	3.0	0.945	3.4	3.0	0.935	2.5	2.9	0.862
Lymph nodes MTV (mL)	9.4	48.7	0.433	8.5	75.1	0.179	11.0	2.6	1.000
Lymph nodes TLG	86.3	279.5	0.301	79.2	432.9	0.104	98.2	11.0	0.862
Lymph nodes SUVmax*	11.3	15.0	0.651	12.4	20.1	0.431	9.3	6.0	0.862
Soft tissue +LFN MTV (mL)	14.0	56.6	0.501	10.1	82.5	0.240	20.6	11.2	1.000
Soft tissue + LFN TLG	103.1	334.2	0.276	88.8	481.9	0.162	127.0	75.7	0.953
Soft tissue + LFN SUVmax*	11.7	15.5	0.573	13.1	20.2	0.464	9.4	7.4	1.000
Lung MTV (mL)	1.6	2.8	0.450	0.1	2.0	0.431	4.2	4.2	0.684
Lung TLG	9.7	9.4	0.519	0.2	2.4	0.431	25.6	21.8	0.684
Lung SUVmax*	2.5	3.6	0.555	0.2	1.0	0.464	6.2	8.1	0.684
Liver MTV (mL)	0.5	1.3	0.483	0.8	1.4	0.766	0.0	1.2	0.521
Liver TLG	1.1	17.0	0.466	1.8	23.7	0.725	0.0	5.2	0.521

Liver SUVmax*	0.1	6.0	0.450	0.2	8.5	0.725	0.0	1.6	0.521
Bone MTV (mL)	0.8	11.3	0.037*	0.0	16.2	0.026*	2.0	2.8	0.684
Bone TLG	1.5	46.5	0.034*	0.0	63.7	0.026*	4.1	16.5	0.599
Bone SUVmax*	0.1	8.7	0.034*	0.0	12.3	0.026*	0.4	2.5	0.599
Other MTV (mL)	2.3	0.9	0.967	1.5	1.4	0.725	3.6	0.0	0.684
Other TLG	16.6	3.0	0.967	13.6	4.8	0.685	21.5	0.0	0.684
Other SUVmax*	2.9	0.8	0.967	3.7	1.2	0.685	1.4	0.0	0.684
Whole body MTV (mL)	19.3	73.2	0.117	12.6	104.0	0.013*	30.5	19.4	0.599
Whole body TLG	129.0	410.2	0.082	99.4	576.5	0.022*	178.3	119.1	0.684
Whole body SUVmax*	14.7	18.6	0.433	14.9	22.0	0.314	14.5	12.7	0.862

NOTE: LFN = lymph nodes; MTV = metabolic tumor volume, SUVmax= maximum standardize uptake value, TLG = total lesion glycolysis.
* The only statistically different means to Mann-Whitney test (*p<0.05*).
